# Metrological Characterization of Low-Cost CO_2_ Sensors for Environmental Monitoring Applications

**DOI:** 10.3390/s26123685

**Published:** 2026-06-09

**Authors:** Ramona Russo, Francesca Rolle, Giuliano Vitali, Francesca Durbiano, Francesca Romana Pennecchi, Stefano Pavarelli, Michela Sega

**Affiliations:** 1 Istituto Nazionale di Ricerca Metrologica (INRiM), 10135 Torino, Italy; r.russo@inrim.it (R.R.); f.rolle@inrim.it (F.R.); f.durbiano@inrim.it (F.D.); f.pennecchi@inrim.it (F.R.P.); s.pavarelli@inrim.it (S.P.); 2 Department of Energy (DENERG), Politecnico di Torino, 10129 Torino, Italy; 3 Department of Agricultural and Food Sciences, University of Bologna, 40126 Bologna, Italy; giuliano.vitali@unibo.it

**Keywords:** low-cost gas sensors, environmental monitoring, atmospheric pollutants, uncertainty evaluation, carbon dioxide

## Abstract

**Highlights:**

**What are the main findings?**

- Low-cost NDIR sensors show high linearity but significant systematic bias before calibration.
- Calibration reduces errors below ~20 µmol/mol; uncertainty is dominated by reproducibility.

**What are the implications of the main findings?**

- Reproducible and scalable approach as a guideline for the characterization and calibration of low-cost sensors for atmospheric gases.
- Calibrated low-cost sensors are suitable for monitoring CO_2_ concentration and fluxes in many contexts.

**Abstract:**

Low-cost sensors are increasingly used in atmospheric monitoring to provide spatially distributed measurements of gas concentrations, often through sensor networks. However, their application is still limited by the lack of metrologically robust characterization procedures. This work addresses a metrological characterization of SCD30 (Sensirion) non-dispersive infrared (NDIR) low-cost sensors for atmospheric carbon dioxide measurements, tested against an NDIR reference analyzer. A dedicated experimental facility and a systematic characterization procedure were developed using a dynamic dilution method in an isolator, covering a concentration range of approximately (350–950) µmol/mol, representative of typical ambient conditions. The analysis focused on sensor performance, calibration functions, uncertainty evaluation, and statistical indicators. Results show that all sensors exhibit good linearity but significant systematic deviations. The uncertainty evaluation highlights reproducibility as the dominant contribution (>85% of the uncertainty budget). The results demonstrate that, after applying calibration, root mean square error (RMSE) and mean absolute error (MAE) are reduced below 20 µmol/mol, demonstrating a substantial improvement in accuracy. The Bland–Altman analysis shows a good agreement between the reference instrument and the low-cost sensors. The proposed methodology provides a robust framework for the metrological evaluation and calibration of low-cost sensors, which can be extended to other atmospheric gases.

## 1. Introduction

Accurate and spatially distributed measurements of atmospheric CO_2_ concentrations have become a central requirement across a broad range of scientific and applied domains, including industrial processes and safety [1,2,3]; Indoor Air Quality [4,5,6,7]; human health and performance research [8,9,10]; transportation and mobility [11,12]; agriculture and greenhouses [13,14,15]; environmental and climate monitoring [16,17,18,19,20].

In the context of climate research, the ability to quantify CO_2_ fluxes at fine spatial and temporal resolution is widely recognized as essential for formulating and evaluating effective mitigation strategies [21]. For this reason, many studies focus on modelling different climate change scenarios [21,22,23]. However, improving the reliability of these models requires highly accurate and widely distributed real-world monitoring data, particularly regarding environmental CO_2_ concentrations. Until now, large-scale CO_2_ measurements have been limited by the high cost of high-precision instrumentation [24]. In recent years, however, low-cost sensors have gained increasing popularity due to advancements in electronics and the Internet of Things (IoT), as they are compact, portable, and energy-efficient devices [24]. These sensors are often integrated with wireless data transmission systems, forming flexible and easily deployable measurement units [25].

Low-cost sensor networks are emerging as promising tools for analyzing local-scale variations in air pollution [26]. Also, in complex urban environments [27], where emissions vary significantly across space and time. A dense network of such sensors can provide valuable insights into carbon fluxes and their sources [25].

Despite economic and operational advantages, their adoption in scientific applications remains constrained by well-documented limitations: sensitivity to temperature, relative humidity, and atmospheric pressure; cross-sensitivity to other gases; and long-term drift associated with component ageing [27]. As a result, raw measurements from low-cost sensors are subject to substantially higher uncertainty than those from reference-grade instruments, which limits their direct use in decision-making and regulatory contexts [25,26].

The technical limitations of low-cost CO_2_ sensors can be addressed through calibration [25]. Since manufacturer-provided calibration is often insufficient or unavailable for many devices, individual sensor calibration and dedicated data processing approaches are frequently required. These procedures may include outlier filtering, quality control strategies, and the implementation of correction models capable of converting raw sensor signals into reliable concentration measurements [25]. Consequently, improving the metrological reliability of low-cost sensing systems remains a key challenge for their effective deployment in large-scale environmental monitoring networks.

The acquisition of reliable and traceable data is therefore essential for scientific and environmental applications involving low-cost sensors, making sensor characterization and calibration fundamental steps in their practical implementation.

Several researchers have already focused on calibrating low-cost sensors: Spinelle et al. (2015) [28], Yasuda et al. (2012) [29] and Hayward et al. (2024) [30] found that such sensors exhibit systematic bias and that calibration must include environmental variables (temperature, relative humidity and pressure). They also recommended periodic recalibration.

Cai et al. [27] performed a long-term field calibration (30 months) of SenseAir K30 sensors co-located with a Cavity Ring-Down Spectroscopy (CRDS) reference instrument in Beijing. The implemented method involves periodic calibration using traceable gas standards and a long-term drift correction procedure, carried out via linear interpolation between known calibration points. The main results concern environmental correction (temperature, humidity and pressure), where the application of the regression model reduced the RMSE from 5.9 µmol/mol to 1.6 µmol/mol and management of drift, which was corrected by stabilizing daily accuracy within a threshold of 5 µmol/mol. In conclusion, the authors recommend a calibration interval of every 3–6 months to maintain the reliability of the results over time.

Mullet et al. [25] presented a network-based calibration based on over 300 SenseAir LP8 sensors. The calibration methodology involved an initial phase in a climate and pressure chamber, followed by a co-location phase in a real-world environment. The approach is based on an empirical parametrization of the effects of temperature and pressure on the sensor’s light emitter. Drift correction takes place under well-mixed atmospheric conditions by correlating sensor data with that of the nearest reference instrument.

Lee et al. [31] proposed a segmented calibration method in which the sensor operating range is divided into multiple intervals, each associated with a specific transfer function. The method was based on the modified progressive polynomial calibration algorithm, which limits the growth of the polynomial degree, thereby reducing computational complexity and improving numerical stability compared to traditional approaches. The results showed a significant reduction in calibration error and demonstrated the effectiveness of the method also in the presence of environmental cross-sensitivity and in embedded systems with limited computational capabilities.

Other recent research trends are concentrating on the development of mathematical and probabilistic frameworks for analyzing sensor behaviour [32,33,34].

Despite this progress, several critical gaps persist in existing literature. First, most published calibration approaches are strongly sensor-specific, relying on empirical correction models tailored to a particular device type, which limits their transferability to other low-cost sensing technologies. Second, and more fundamentally, the metrological foundations of calibration procedures are rarely addressed explicitly: traceability to recognized standards, rigorous uncertainty quantification, and detailed descriptions of the controlled experimental infrastructure required for reproducible calibrations are largely absent from the literature. In the case of CO_2_ sensors, their calibration should cover the objectives of the Global Atmosphere Watch Programme (GAW) of the World Meteorological Organization (WMO), which require a precision of 0.1–0.2 µmol/mol for global monitoring [35].

The present study addresses these gaps by proposing a metrologically traceable and reproducible calibration procedure for low-cost gas sensors, with particular emphasis on uncertainty quantification, traceability to recognized standards, and controlled experimental conditions. Unlike many previous studies focused mainly on data-driven correction models, this work provides a detailed description of the experimental infrastructure and calibration workflow developed at the QM02 laboratory of the Italian National Institute of Metrological Research (INRiM). The proposed methodology is scalable and can be extended to larger arrays of low-cost sensors, as well as adapted for the analysis of various other gas mixtures, such as [36,37].

## 2. Materials and Methods

The technology evaluated in this study was Non-Dispersive Infrared (NDIR) spectroscopy, a technique that has been recommended by the World Meteorological Organization (WMO) for its suitability in portable instrumentation [38]. It is also considered one of the most commercially available types, as well as a robust and selective detection technique with a very low cross-selectivity [38].

The experimental activities employed a high-accuracy CO_2_ analyzer as the reference instrument. This instrument had been previously calibrated according to INRiM internal procedures using certified gas mixtures.

The reference device is an LI-COR 850 analyzer (LI-COR, Lincoln, NE, USA) [39], based on NDIR technology, with a measurement range from 0 to 20,000 µmol/mol of CO_2_ and a nominal (manufacturer-specified) accuracy of ±1.5% of the reading. The expanded uncertainty, as evaluated at INRiM, ranges from 0.8 µmol/mol (at the lowest certified concentration of 200 µmol/mol) to 3.6 µmol/mol (at the highest certified concentration of 900 µmol/mol). Within this work, this instrument is defined as the primary reference instrument (LI-cor1).

A second instrument of the same model, with identical nominal specifications, was also employed and is referred to as the secondary instrument (LI-cor2). Although not calibrated, this analyzer has been extensively used in previous laboratory activities, where its measurements were verified to be fully consistent with those of LI-cor1. For this reason, LI-cor2 was used as an additional control to monitor CO_2_ concentration within the isolator during the experiments.

For the low-cost sensors, the choice of Sensirion SCD30 (Sensirion AG, Stäfa, Switzerland) [40] was made on the basis of several considerations and comparisons of features, some of which have been reported in Table 1: cost, accuracy and stability were the primary scores, while size was not considered a relevant aspect; temperature *T* and relative humidity *RH* were also considered. The model SCD41, though smaller and more efficient than SCD30, has a lower accuracy; therefore, the latter has been preferred. The true competitor of SCD30 is SenseAir S8 (Asahi Kasei Group, Delsbo, Sweden, Italy), but it does not include *T/RH* sensors and is more complex to connect, as it is mainly designed for fixed systems. The cost of the sensors ranges from €15 to €120.

The SCD30 is characterized by a dual-channel NDIR configuration, designed to ensure good long-term stability and accurate CO_2_ measurements; the presence of two measurement channels enables compensation for drift and temporal variations, thereby improving reliability compared to simpler sensor designs. According to the manufacturer’s data sheet, the sensor measures CO_2_ concentrations typically in the range from 400 µmol/mol to 10,000 µmol/mol, with a vendor-specified accuracy of ±30 µmol/mol + 3% of the measured value and a response time of approximately 20 s. An important feature of the module is the integration of *T* and *RH* sensors, which are not merely auxiliary outputs but are actively used to compensate for the CO_2_ measurement. Consequently, the overall data quality also depends on the correct operation and placement of the sensor within the environment, as factors such as local heating effects or non-uniform airflow may introduce indirect measurement errors.

Three SCD30 sensor modules were used and later identified as sen1, sen2, and sen3.

The isolator chamber was developed in response to the growing demand for the simultaneous testing of many sensors in their ‘bare’ configuration (i.e., limited to the sensing element without any housing), as required by the increasing use of low-cost sensors. The isolator made of transparent poly(methyl methacrylate) (PMMA) with dimensions of (80 × 80 × 80) cm (Montepaone, San Mauro Torinese, Italy) was designed at INRiM (in Supplementary Materials Figure S1). The transparent structure enables continuous visual monitoring of the internal conditions during experimental tests. The isolator is equipped with two openings of (60 × 30) cm, positioned on adjacent sides, to facilitate the insertion and positioning of instrumentation. It is also fitted with a temperature control system based on a chiller, enabling operation within a range from −25 °C to 150 °C (from the datasheet) [41]. The chiller plate is installed at the bottom of the isolator and covered with a perforated plate made of the same material as the enclosure to promote adequate air mixing within the test volume. The chiller unit is in the lower part of the supporting structure, consisting of a steel platform equipped with wheels, designed to facilitate the movement of the entire system.

Prior to use, the isolator underwent a characterization phase. In particular, a decay test was performed using CO_2_ as a tracer gas, demonstrating that, when sealed, the system exhibits high airtightness, with an air exchange rate equal to 0.0012 vol/h. The internal temperature uniformity was also assessed under ambient conditions (i.e., without active chiller control), using three Pt100 sensors previously calibrated according to internal laboratory procedures. The maximum temperature difference recorded among the three measurement points was ~0.002 °C, confirming a high level of thermal uniformity within the isolator.

To promote uniform gas distribution within the isolator, a 15 V fan was employed.

Pure nitrogen (N_2_) (grade 6.0), supplied by Air Liquide (Milano, Italy), was used as zero gas, and a gas mixture of CO_2_ in N_2_ with a concentration of 80,325 µmol/mol, gravimetrically prepared at INRiM following the prescriptions of the International Standard ISO 6142-1 [42], was employed for dynamic mixing inside the isolator.

The gas flow was controlled by a calibrated Mass Flow Controller (MFC) (MKS Instruments, Andover MA, USA), with a full scale of 2000 standard cubic centimetres per minute (SCCM).

### 2.1. Experimental Set-Up

The two high-quality instruments and the three low-cost sensors were installed inside the isolator, which was operated under uncontrolled ambient conditions and used as a mixing chamber for dynamic dilutions. The two gas cylinders were connected to a selection valve, allowing the choice of the gas to be injected. The selected gas stream was then regulated by the MFC and introduced into the isolator.

By injecting controlled amounts of gas, a target concentration was reached within the isolator. In particular, CO_2_ and N_2_ flows were adjusted to respectively increase or decrease the CO_2_ concentration, enabling the generation of predefined concentration levels.

To ensure proper gas homogenization within the isolator, two high-quality reference instruments were employed, providing an extra measurement and enabling cross-validation of the concentration levels inside the chamber.

**Figure 1:**
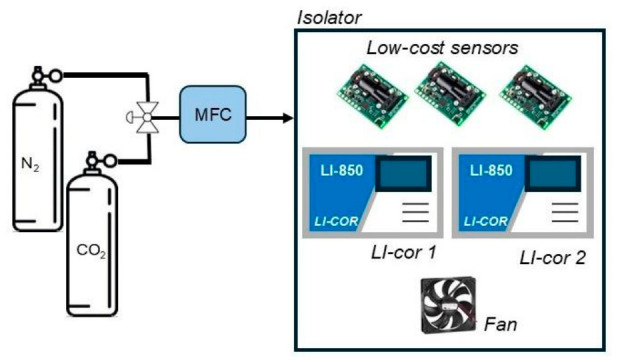
Schematic representation of the experimental set-up.

A schematic representation of the experimental setup for the characterization of the low-cost sensors is shown in Figure 1.

The three low-cost sensors were characterized by comparison with the reference instrument, LI-cor1, over a concentration range spanning approximately between (350–950) µmol/mol, representative of typical ambient atmospheric conditions. During the tests, both the reference instrument (LI-cor1) and the low-cost sensors continuously recorded measurements: the LI-cor1 every 5 s and the low-cost sensors approximately every 20 s.

**Figure 2:**
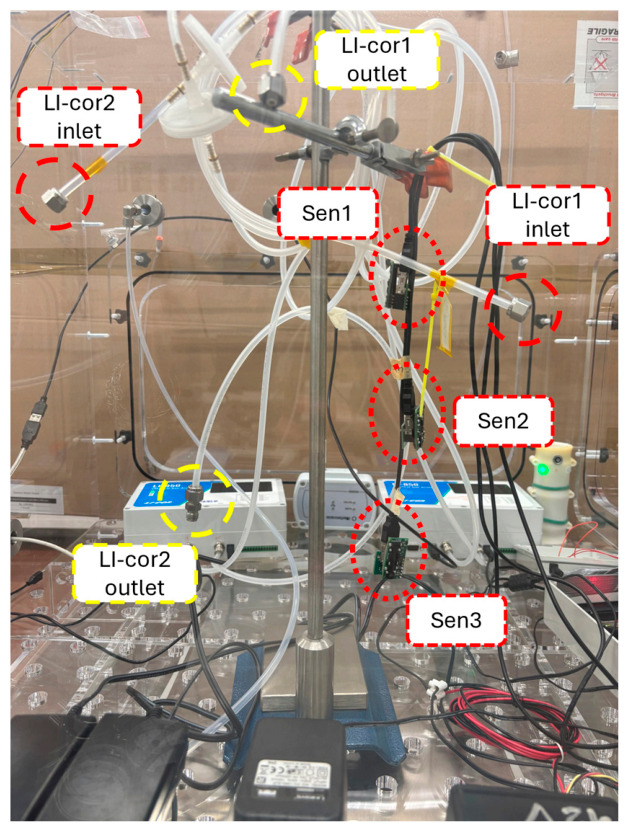
Photo of the experimental set-up realized in the isolator.

Figure 2 shows the inlets and outlets of the reference sensors and the position of the low-cost sensors inside the isolator.

### 2.2. Calibration Methods

In this study, two methods for calibration of the low-cost sensors were considered.

According to the first one, the sensor response is calibrated using a step function that is a piecewise constant function, assuming constant values over predefined intervals and exhibiting discontinuities at the interval boundaries [43]. This kind of calibration procedure can be applied to all the sensors, regardless of their specific characteristics and even in the absence of linear behaviour. The result of this method is a table reporting the measurement results of the low-cost sensor together with their corresponding errors, for which they need to be corrected, and uncertainties associated with those errors. This information can then be used to correct subsequent measurements.

The second method involves a more in-depth analysis and can only be applied if the sensor exhibits a functional dependence on the sensor readings with respect to the reference values. In this case, a polynomial curve is typically fitted to the data according to some appropriate regression method. In the present study, the Weighted Total Least Squares (WTLS) regression was applied because it is able to account for uncertainty in both variables [44].

#### 2.2.1. Low-Cost Sensors Correction and Associated Uncertainty

Since low-cost sensors frequently deviate from linear behaviour, we propose a step approach that can be effectively applied to any sensor under test, regardless of its specific characteristics.

Measurement points, between 350 µmol/mol and 950 µmol/mol, were selected as representative of the concentration range under analysis. To account for the sensor error at each point, a correction associated with each sensor was evaluated on the basis of the readings acquired in a 10 min time interval corresponding to a stable CO_2_ concentration. In order to ensure a metrologically valid comparison and to reduce the impact of high-frequency measurement noise, a time-averaging of the readings was taken.

For each 10 min interval, the reference CO_2_ concentration was computed as the arithmetic mean CO_2,ref_ of the reference instrument readings. Similarly, the sensor output was estimated as the mean CO_2,sen_ of the measurements made in the same 10 min interval by each low-cost sensor. The measurement error *ε* was defined as:

(1) $$\varepsilon \;=\;{CO}_{2,sen}{\;-\;CO}_{2,ref}$$

This definition allows the identification of systematic deviations, with positive values indicating overestimation and negative values indicating underestimation by the sensor, respectively.

The uncertainty $u_{\varepsilon }$ associated with the error *ε* was evaluated following a bottom-up approach consistent with the GUM framework [45], i.e., by combining the main sources of uncertainty affecting both the reference instrument and the low-cost sensor measurements, which are:

- Calibration of the reference instrument (*u*_cal_): uncertainty term that ensures the traceability of the reference sensors:
  (2) $$u_{cal}=U(d)/2\;$$
  where *U*(*d*) is the expanded uncertainty derived from its calibration certificate (other uncertainty contributions such as repeatability and reproducibility were negligible for the reference sensor measurements).
- Resolution of the low-cost sensor (*u*_res_): the uncertainty due to the resolution of the sensors was evaluated assuming a rectangular probability distribution:
  (3) $$u_{res}=\delta /2\sqrt{3}$$
  where δ represents the sensor resolution as specified in the manufacturer’s datasheet.
- Repeatability of the low-cost sensor (*u*_repeat_): the repeatability component was evaluated as the standard deviation of the mean of repeated measurements within each 10 min interval, performed under nominally identical conditions:
  (4) $$u_{repeat}=s_{sen}/\sqrt{n_{sen}}$$
  where *s*_sen_ is the standard deviation of the sensor measurements in each 10 min interval, and *n*_sen_ is the number of measurements.
- Reproducibility of the low-cost sensor (*u*_reprod_): the reproducibility component was evaluated as the standard deviation of three independent measurements repeated at the same concentration with a minimum of 4 h between repetitions. The corresponding uncertainty contribution was calculated as:
  (5) $$u_{reprod}=s_{{CO}_{2,sen}}$$
  where $s_{{CO}_{2,sen}}$ is the standard deviation of the three mean responses obtained at corresponding 10 min intervals in the three independent measurement repetitions.
  The combined standard uncertainty $u_{\varepsilon }\;$ associated with the measurement error was hence obtained by applying the law of uncertainty propagation to model (1), taking into account all the contributions reported above:
  (6) $$u_{\varepsilon }=\sqrt{\left(u_{cal}^{2}+\;u_{res}^{2}+{u\;}_{repeat}^{2}+\;u_{reprod}^{2}\right)}$$
  where, in a conservative approach, the maximum repeatability uncertainty among the repetitions was employed in order to account for the worst-case variability.

Finally, the expanded uncertainty *U* was calculated to define the confidence interval of the measurement at a desired level of confidence:

*U = k × u_ε_* (7)

where *k* is the coverage factor. In compliance with the GUM guidelines, a factor of *k* = 2 is used, providing a confidence level of approximately 95%, assuming a normal distribution is associated with the measurand.

All uncertainty contributions not explicitly included in the analysis were considered negligible for the purposes of this study.

Eventually, a table was produced in which all the components of the uncertainty budget for the calibration are reported. The table contains the values read by the low-cost sensor, the corresponding errors and the uncertainties associated with the errors. Based on the experimental results, a correction table can identify the concentration intervals, and the corresponding correction associated with each interval can be applied to subsequent measurements. As a consequence, the resulting calibration model can be interpreted as a step function.

The correction to apply to future readings, provided by the low-cost sensors when used in real-world applications, will be performed according to Equation (8):

(8) $${CO}_{2,corr}={CO}_{2,sen}+\varepsilon \;$$

(9) $$U({CO}_{2,corr})=2\times \left(\sqrt{{\left(s_{sen}/\sqrt{n}\right)}^{2}+{u_{\varepsilon }}^{2}}\right)$$

where ${CO}_{2,corr}$ is the corrected value, ${CO}_{2,sen}$ is the rough reading of the low-cost sensor, ε is the error reported in the calibration table at a concentration value equal to or close to ${CO}_{2,sen}.$ The expanded uncertainty $U\left({CO}_{2,corr}\right)$ associated with the correction is obtained according to Equation (9) on the basis of the repeatability uncertainty$\;\left(s_{sen}/\sqrt{n}\right)$ of the low-cost sensor measurements obtained when using the calibrated sensor in the field, and the error uncertainty$\;u_{\varepsilon }$ reported in the table.

Measured concentration values, even when different from the specific calibration points, will fall within concentration intervals previously defined based on the experimental calibration results and reported in the correction table. Therefore, each measurement can be corrected using the error value and the associated uncertainty corresponding to the interval in which the measured concentration lies.

#### 2.2.2. Weighted Total Least Squares (WTLS) Regression

If the sensor showed a functional dependence of the readings with respect to the reference instrument readings, a proper analysis curve could be determined by fitting the data. In this case, the calibration curve was obtained by implementing a Weighted Total Least Squares (WTLS) regression method, which is able to take into account the uncertainties associated with both dependent and independent variables. The regression was performed using the CCC software (v2.0) [46]. An analysis curve was fitted by providing as input data to the software the measurements obtained from the low-cost sensors (average of the three repetitions), i.e., the independent *x* values, and the reference measurements (average of the three reference readings) as the dependent *y* values. As input to the software, covariance matrices associated with *x* and *y* values were provided, which were diagonal matrices reporting on their diagonal the combined squared uncertainties of CO_2,sens_ and CO_2,ref_, respectively. For all sensors, a linear regression model (*y = a + bx*) was adopted. The CCC software provided the estimates of the coefficients *a* and *b*, together with their associated uncertainties and covariance, the fitted values on the curve and the associated uncertainties.

Once the calibration parameters were estimated, the analysis curve could be used to estimate the true gas concentration from a new sensor reading taken in the field according to the following equation:

(10) $${CO}_{2,corr}=a+\;b\times {CO}_{2,sen}\;$$

The associated standard uncertainty is evaluated according to:

(11) $$u^{2}\left(y\right)=u^{2}\left(a\right)+u^{2}\left(b\right)\times {{CO}_{2,sen}}^{2}+b^{2}\times u^{2}{(CO}_{2,sen})+\;2u(a,b)\times \;{CO}_{2,sen}$$

where *u*(*a*) and *u*(*b*) are the standard uncertainties associated with the intercept and slope coefficients, respectively, *u*(*a*,*b*) is their covariance, and *u*(CO_2,sen_) is the standard uncertainty associated with the sensor reading. The covariance term is due to the statistical correlation between the fitted regression parameters.

### 2.3. Statistical Analysis and Performance Metrics

The multi-point/tabular calibration performance was validated through statistical analysis including RMSE, MAE, Bland–Altman analysis, and coefficient of determination (*R*^2^), whereas the WTLS fitting calibration validity was assessed through the normalized chi-square statistic and the plot of residuals.

#### 2.3.1. Root Mean Square Error (RMSE)

The RMSE is used to quantify the overall deviation between each sensor and the reference, the metric being applied to the average values over 10 min time intervals:

(12) $$RMSE\;=\sqrt{\left(\frac{1}{n}\sum {\left({CO}_{2,sen}-\;{CO}_{2,ref}\right)}^{2}\right)}$$

where *n* is the total number of readings taken in the entire range of concentrations analyzed.

#### 2.3.2. Mean Absolute Error (MAE)

The MAE provides a robust estimate of the average absolute deviation, considering the average values over 10 min time intervals:

(13) $$MAE\;=\frac{1}{n}\sum \left|{CO}_{2,sen}-{CO}_{2,ref\;}\right|$$

where *n* is the total number of readings taken in the entire range of concentrations analyzed.

#### 2.3.3. Bland–Altman Analysis

The Bland–Altman plot is used to describe agreement between two quantitative measurements by creating limits of agreement (LoA). These statistical boundaries are determined using the mean (D) and standard deviation (*s*) of the differences between the two measurements using a graphical method [47]. The resulting graph (in Section 3.2) is a scatter plot with the *y*-axis displaying the difference ∆CO_2_ (CO_2,ref_ − CO_2,sen_) between the measurements and the *x*-axis indicating the mean of these values ${CO}_{2}$ ((CO_2,ref_ + CO_2,sen_)/2). Essentially, the difference in the two paired measurements was plotted against their average. Bland–Altman analysis suggested that 95% of the data points should fall within ±1.96 times the standard deviation of the mean difference.

#### 2.3.4. Coefficient of Determination (R^2^)

The coefficient of determination (*R*^2^), i.e., the squared correlation [48], was adopted to study the linearity of the dependence of the sensor response on the reference measurements. An *R*^2^ value close to 1 indicates a strong linear dependence between the sensor and reference.

#### 2.3.5. Normalized Chi-Square Statistic

When the regression analysis described in Section 2.2.2 is performed, the CCC software provides the normalized chi-square statistic, (*χ*^2^/(*n* − *p*)), where *χ*^2^ is the weighted sum of squared residuals and (*n* − *p*) is the difference between the number of observations (n) and the number of fitted parameters (p), corresponding to the degrees of freedom of the model. A value of the normalized chi-square statistic close to 1 indicates that the agreement between the observations and the fitted model is consistent with the input uncertainties.

## 3. Results

As explained in Section 2, this research utilized two high-quality instruments and three low-cost sensors. The two reference instruments provide highly comparable results (as demonstrated in Supplementary Materials Figure S2). Consequently, LI-cor2 will not be considered for the study, while all subsequent analyses were conducted utilizing LI-cor1 as the primary instrument, already calibrated.

The acquisition of data was conducted over a period of more than two consecutive weeks, with the aim of generating a dataset that was statistically significant and representative of the operational conditions.

The performance of the low-cost CO_2_ sensors was evaluated by comparing their response with the reference instrument across multiple concentration levels within the ~350 µmol/mol and ~950 µmol/mol range reported in Table 2.

In order to investigate the behaviour of the sensors even below the lower limit declared by the manufacturer, measurements were performed at 348 µmol/mol, a value slightly lower than the typical atmospheric concentration but still realistic in extremely positive environmental conditions.

For each concentration level, three independent measurements were taken (repetitions I, II, III), thereby constructing increasing and decreasing concentration scales within the closed isolator, with the objective of minimizing external contamination and ensuring controlled conditions.

The environmental parameters within the isolator were not controlled; rather, they were the subject of continuous monitoring. Throughout the experimental campaign, *T* and *RH* were maintained at levels typical of an indoor environment. *T* values in the range (24–27) °C and *HR* values between (24–45)% were recorded. The environmental parameters measured during the 10 min intervals analyzed are shown in Supplementary Materials Figure S3, with temperatures ranging from 26.44 °C to 24.63 °C and relative humidity from 41% to 28%. These sensors were previously tested in a climatic chamber in a temperature range between 15 °C and 30 °C and relative humidity between 30% and 60%. The results showed no variations in CO_2_ measurement resulting from changes in these parameters.

**Figure 3:**
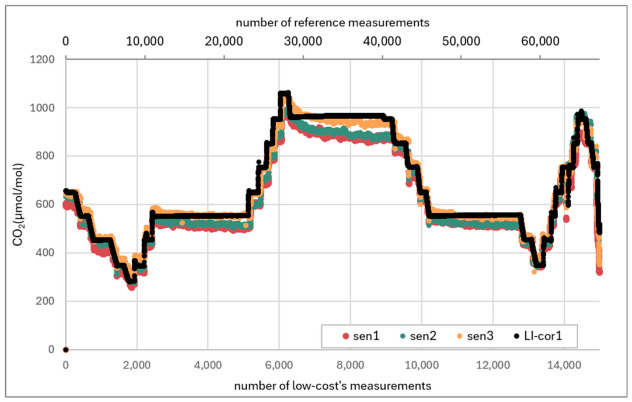
CO_2_ measurements were made by the three low-cost sensors and the reference instrument LI-cor1 during this research.

As demonstrated in Figure 3, all sensors exhibited a similar trend to the reference instrument across the entire concentration range. Even though the concentration of the substance under investigation was at its lowest recorded level of 348 µmol/mol, which is outside the range declared by the manufacturer, the sensors still demonstrated a consistent response, albeit with greater dispersion.

In this study, a 10 min analysis for each repetition for each concentration was considered. In Supplementary Materials, Figure S4 presents, as an example, the 10 min analysis for the sen3 sensor. It is evident that the reference instrument demonstrates elevated stability, characterized by overlapping curves across repeated measurements, while the low-cost sensors exhibit greater dispersion. Figure S5 illustrates the mean value of the three replicates for each sensor. The graph demonstrates that: sen1 shows an increasing offset with concentration and exhibits the greatest deviation from the reference; sen2 demonstrates good overall agreement, with more marked deviations at 649 µmol/mol and 952 µmol/mol; sen3 is the closest to the reference across the entire range analyzed. It is also interesting to note that, comparing the low-cost sensors among each other, their respective offsets show the same trend: sen1 measurements are invariably smaller than those from sen2, which, in turn, are smaller than those from sen3.

### 3.1. Uncertainty of the Correction

Table 3 presents the mean values of the three repetitions, calculated over the 10 min analysis intervals for each concentration. The corresponding ε errors, which quantify a systematic deviation from the reference, are also reported. The error exhibited variability across the concentration range that was investigated, thus indicating the presence of both offset and a potential concentration-dependent bias. In particular, sen1 shows an increasing systematic underestimation with concentration (up to −73.58 µmol/mol at the highest concentration of ~952 µmol/mol), indicating a gain error; sen2 exhibits intermediate behaviour, with both positive and negative errors, suggesting a combination of offset and nonlinearity. The sen3 model demonstrated the highest degree of accuracy, with error ranges from approximately +21 µmol/mol to −13 µmol/mol, and a total variation of 34 µmol/mol.

The initial phase of the analysis consisted of determining the combined uncertainty, which integrated all the components discussed in Section 2.2.1. The experimental results obtained are presented below as an example of the application of the analysis protocol developed at INRiM for the metrological calibration of low-cost CO_2_ sensors.

**Figure 4:**
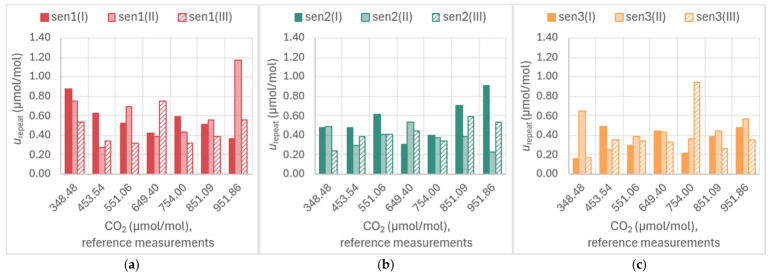
Repeatability uncertainty was analyzed for all three repeats for the entire CO_2_ range analyzed: (**a**) repeatability uncertainty of sen1; (**b**) repeatability uncertainty of sen2; (**c**) repeatability uncertainty of sen3.

Repeatability analysis confirmed that the intrinsic variability at each 10 min interval is low (Figure 4).

Specifically, the maximum repeatability standard deviations recorded for the sen1, sen2, and sen3 sensors were 6.21 µmol/mol, 4.70 µmol/mol, and 5.10 µmol/mol, respectively. For the sen1 and sen2 sensors, the variability was observed at the highest concentration level (951.86 µmol/mol), while for the sen3 sensor, the highest standard deviation was found at 754.00 µmol/mol.

The combined uncertainty is thus dominated by the reproducibility component (*u*_reprod_), which in many cases represents over 85% of the total uncertainty budget (Figure S6). This finding indicates that the primary constraint of low-cost sensors is not resolution or short-term repeatability, but rather stability between independent measurements.

The contributions of *u*_cal_ and *u*_res_ are the same for the three sensors and, at the end, are negligible in the combined uncertainty.

Adopting a conservative approach (maximum repeatability value) is known to ensure metrological robustness, although it can lead to a slight over-evaluation of uncertainty.

After all the analyses have been carried out, the uncertainty budget tables for each sensor were developed and are reported in Table 4, Table 5 and Table 6.

For sen1, the *u*_reprod_ ranges from 3.80 µmol/mol to 31.18 µmol/mol. For sen2, the dispersion of the mean is found to be significantly amplified at specific concentration levels, with a maximum of 49.96 µmol/mol observed at a concentration of 951.86 µmol/mol. For the sen3, the maximum *u*_reprod_ value is 26.59 µmol/mol, which is a very good value for a low-cost sensor.

For all the low-cost sensors, *U* exhibits a pronounced concentration dependence driven by the anomalous outlier session, reaching ~62 µmol/mol and ~100 µmol/mol at 851 and 951 µmol/mol, respectively. Sen3 sensor shows a more regular behaviour, with U ranging from ~5 µmol/mol to ~53 µmol/mol (Figure S7).

In Supplementary Materials, Figure S7 presents the behaviour of error ε as a function of reference concentration, specifically, it shows the mean error among repetitions (I, II and III) and the expanded uncertainty band ±*U*. This representation allows for the simultaneous visualization of systematic bias, random variability, and the concentration dependence of the error. Sen1 shows systematic and concentration-dependent underestimation, with ε ranging from −21.34 µmol/mol at 348 µmol/mol to −73.58 µmol/mol at 952 µmol/mol.

For practical applications, we therefore recommend defining in advance the expected concentration range of the target gas, in order to construct the correction table within the most appropriate operating range. In this case, the correction table is Table 7, where, after the analysis of the uncertainty budget tables, the concentration interval was decided to be every 100 µmol/mol. Once the correction table has been established, the sensors can be employed for measurements, and the recorded values should be corrected according to Equation (8) reported in Section 2.2.1.

**Figure 5:**
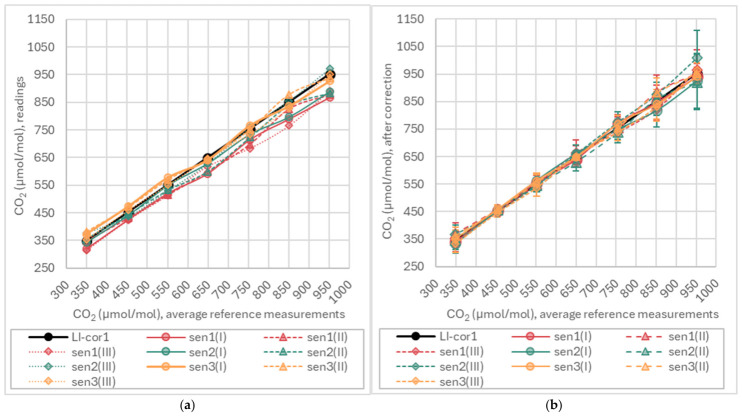
Readings of CO_2_ of the reference instrument and low-cost sensors: (**a**) before correction; (**b**) after correction with the expanded uncertainties (*k* = 2).

When the entire measurement chain is taken into consideration, all three sensors demonstrate adequate linearity, thus enabling the implementation of a linear transfer function for calibration purposes (Figure 5a). The correction, which is the sum of the average error value obtained from the three readings, is applied to each reading, with the result that all readings are significantly improved (Figure 5b).

### 3.2. Statistical Performance Metrics

In order to perform a quantitative evaluation of sensor performance, the present study employed a number of statistical indicators that have been frequently utilized within the existing literature. These indicators are outlined in Section 2.3.

**Figure 6:**
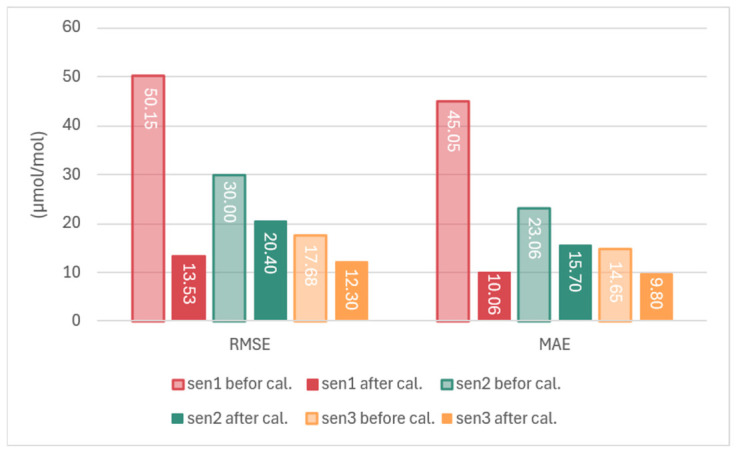
Primary performance indicators (RMSE, MAE) for the three sensors, both before and after calibration.

Figure 6 provides a summary of the primary performance indicators for the three sensors, conducted on all 21 data points (7 concentration levels × 3 repetitions) for each sensor, both before and after calibration. In the pre-calibration phase (light columns), the sensors exhibit RMSE values ranging from ~50 µmol/mol to ~18 µmol/mol, while the MAE ranges from ~40 µmol/mol to ~15 µmol/mol. The discrepancy between the RMSE and the MAE at this stage indicates that the error is not purely stochastic but is dominated by a significant systematic component (bias), as previously observed in the offset analysis. In particular, sen1 demonstrates the highest initial error, consistent with the negative bias of approximately −27 µmol/mol that was detected during the characterization process. The application of the calibration model resulted in a significant improvement in data quality. Following calibration (darker columns), the RMSE and MAE were reduced to exceptionally low values, falling below ~20 µmol/mol.

**Figure 7:**
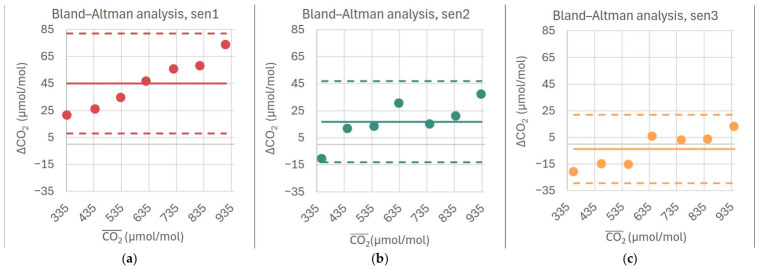
Plot of differences between low-cost sensor average measurements and reference average measurements vs. the mean of the two measurements (data from Table 4, Table 5 and Table 6) (solid circles), highlighting the LoA (dashed lines) and the bias (continuous line): (**a**) Bland–Altman analysis of sen1 (red); (**b**) Bland–Altman analysis of sen2 (green); (**c**) Bland–Altman analysis of sen3 (yellow).

The Bland–Altman plots reported in Figure 7 highlight the agreement between the measurements provided by the three low-cost sensors and the reference instrument over the investigated concentration range. All three low-cost sensors exhibit acceptable agreement with the reference instrument, since all data points fall within the LoA. For sensor 1 (Figure 7a), the differences ∆CO_2_ are consistently positive and increase with increasing CO_2_ concentration, suggesting the presence of a systematic proportional bias. It presents the highest bias (45.01 µmol/mol) and the biggest LoA (37.05 µmol/mol) of the three sensors. Sensor 2 (Figure 7b) shows the best agreement among the three sensors because the data points are more symmetrically distributed around the bias line. For sensor 3 (Figure 7c), the bias is very small (−3.80 µmol/mol), and the differences are predominantly negative at lower concentrations and tend to increase towards positive values as the concentration rises.

The coefficient of determination (*R*^2^) for the three sensors demonstrated a well-defined linear response within the tested range, with values highly comparable and very close to 1, indicating a strong linear correlation and therefore a good ability of the sensor to follow concentration variations.

- Sen1: *R*^2^ = 0.9998
- Sen2: *R*^2^ = 0.9982
- Sen3: *R*^2^ = 0.9994

### 3.3. Application of Regression Analysis

The sensors demonstrated a good linearity, so it was possible to fit the straight line (*y = a + bx*) analysis curves. These curves were constructed using the CCC software, which employs the WTLS method with known uncertainties. The CCC software provided the estimates of the coefficients *a* and *b*, together with their associated uncertainties and the covariance *u*(*a*,*b*), and calculated the normalized chi-square statistic (*χ*^2^/(*n − p*)), all reported in Table 8.

**Figure 8:**
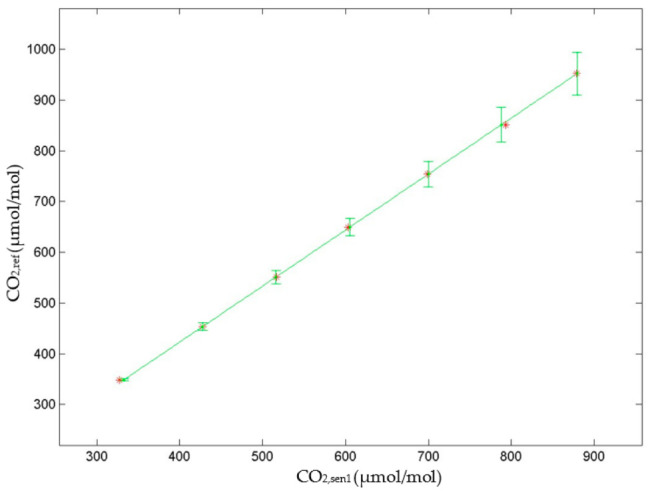
Analysis curve for sen1 with associated uncertainty, elaboration results plot produced by the CCC software for a linear WTLS regression. Red asterisks represent the measured concentration, and the errors bars are the expanded uncertainties (*k* = 2) for the regression.

Figure 8 shows the analysis curve obtained for Sen1 as an example; the other calibration curves are in the Supplementary Materials (Figures S8 and S9). The experimental points (red stars in the figure) exhibited a linear behaviour over the investigated concentration range. The fitted values (green points in the figure) have relatively small uncertainty bars compared to the full-scale span.

The fitted model adequately reproduced the experimental data, as also confirmed by the low normalized chi-square value and by the residual analysis reported in the Supplementary Materials (Figures S10–S12). The results show distinct behaviours across sensors: sen1 exhibits the highest accuracy, with residuals tightly and randomly distributed around the zero line, confirming a robust linear fit without systematic bias. In contrast, sen2 shows a significantly higher data dispersion, indicating lower experimental precision or higher noise, although the errors still fluctuate randomly above and below the baseline. Finally, sen3 reveals a clear systematic underestimation, as almost all residuals are consistently positive. Incidentally, it was checked that a parabolic fit would behave better, leading to a smaller normalized chi-square value.

## 4. Discussion

Although low-cost NDIR CO_2_ sensors are increasingly employed for environmental monitoring applications, several studies have highlighted limitations related to sensor drift, concentration-dependent bias, environmental cross-sensitivity, and non-linear response behaviour, particularly under varying temperature and humidity conditions [24,27,32,49]. Consequently, accurate calibration procedures remain essential to ensure reliable measurements.

This study aims to provide guidelines for the correct calibration of low-cost sensors, and therefore, the analyzed sensors served as an example to explain the calibration procedure. The experimental results indicate that, prior to any correction, the NDIR technology under analysis exhibits inconsistent performance. The sen3 sensor demonstrated the optimal raw performance, while sen1 exhibited the most problematic behaviour, characterized by a marked gain error.

It is observed that the CO_2_ detection range of the low-cost sensors analyzed, as indicated in the datasheet specifications (starting from 400 µmol/mol), is fully consistent with current global atmospheric concentrations. According to data from the NOAA Global Monitoring Laboratory (2026), the global weekly average concentration has reached approximately 428.89 µmol/mol [50]. Therefore, the sensors tested are suitable for monitoring the environmental background and variations in this caused by anthropogenic interference typical of urban areas.

The management of sampling frequencies represented a critical aspect of the experimental design. The decision to utilize the reference instrument at 5 s intervals was essential for the effective monitoring of the dynamic dilution occurring in the isolator with high resolution. Conversely, for the low-cost sensors, a sampling rate of 20 s was necessary to mitigate the influence of electronic background noise and the instrument’s internal resolution.

The evaluation of the expanded uncertainty *U*, carried out using a bottom-up approach consistent with the GUM framework [45], revealed that the dominant limitation is not related to resolution *u*_res_ or reference calibration *u*_cal_, contributions which are negligible. The primary source of uncertainty is inter-session reproducibility *u*_reprod_, defined as the sensor’s capacity to maintain a consistent response across independent measurement cycles. This evidence suggests two research priorities for future studies: firstly, the characterization of the drivers of variability (thermal drift, humidity, warm-up times); and secondly, an increase in the number of replicates to make the evaluation of *u*_reprod_ statistically more robust.

The proposed correction tables are based on the assumption, also shared by Lee et al. [31], that, due to the limited linearity typically exhibited by low-cost sensors, their response can be analyzed over discrete concentration ranges rather than through a single global calibration function. Consequently, the calibration procedure can be interpreted as a stepwise correction approach, in which specific correction values are associated with predefined concentration intervals derived from the experimental calibration results.

The analysis of the linear regression model represents a rapid and effective approach for assessing the linearity of low-cost sensors. However, we recommend using this analysis as a preliminary evaluation to determine whether a more rigorous calibration approach, such as the WTLS regression method, can be appropriately applied. In this work, the WTLS-based analysis curve estimation is therefore presented as a supplementary, non-mandatory analysis, since the proposed point-by-point calibration method, when properly performed, is already sufficiently robust for correcting measurements obtained from low-cost sensors.

The substantial decrease in RMSE and MAE (Figure 6) following calibration serves to confirm the efficacy of the method. The post-calibration residuals were significantly reduced and did not show evident systematic behaviour. The RMSE values obtained in this study are consistent with the data reported in the existing literature: Mueller et al. [25] report values ranging from 8 µmol/mol to 12 µmol/mol over extended periods (19–25 months). Conversely, Cai et al. [27] achieved reductions from 5.9 µmol/mol to 1.6 µmol/mol through the implementation of drift stabilization techniques. The minor discrepancies observed in the present study in comparison to the findings of these authors are primarily attributable to the distinct construction tolerances associated with the various generations of sensors, rather than to the validity of the employed analytical methodology. Compared with previous studies based mainly on empirical or machine-learning correction approaches [51,52], the proposed methodology prioritizes metrological traceability and reproducibility of the calibration process. Although the achieved accuracy is comparable to that reported in the recent literature, the main advantage of the proposed approach lies in the detailed characterization of uncertainty sources and in the use of controlled calibration conditions. On the other hand, the methodology requires dedicated laboratory infrastructure and controlled gas mixtures, which may limit its immediate applicability in low-resource operational contexts.

Although the post-calibration performance achieved in this study is suitable for urban mapping and high-spatial-resolution monitoring applications, the investigated low-cost sensors still do not satisfy the compatibility goals defined by the WMO Global Atmosphere Watch programme for global atmospheric monitoring (0.1–0.2 µmol/mol) [35]. This limitation reflects the intrinsic technological constraints of current budget-class NDIR sensors, including sensor drift, environmental sensitivity, and inter-device variability. Nevertheless, after metrological calibration, these devices can still represent valuable tools for the analysis of local CO_2_ gradients and urban-scale emission patterns.

## 5. Conclusions

This study presents a fully reproducible and metrologically traceable calibration procedure for low-cost gas sensors. This procedure is demonstrated using non-dispersive infrared (NDIR) CO_2_ sensors (SCD30, Sensirion) as a representative case. The proposed tabular methodology is independent of the type of sensor and can be applied to any low-cost sensor, regardless of its specific response characteristics. For sensors exhibiting linear behaviour, additional guidelines for determining calibration and analytical curves using CCC software are also provided.

Before calibration, all sensors exhibited systematic deviations from the reference instrument, including both offset and concentration-dependent bias. Despite these deviations, all sensors demonstrated good linearity (*R*^2^ close to 1), enabling the use of simple linear calibration models. The application of these models resulted in a substantial improvement in performance, reducing RMSE and MAE values below 20 µmol/mol. The convergence of these two indicators after calibration confirms that the dominant systematic component was effectively removed, leaving mainly random variability. Bland–Altman analysis showed that all sensors fall within the limits of agreement with the reference instrument.

Uncertainty analysis revealed that the dominant contribution is due to reproducibility between independent measurements performed in different measurement cycles. In many cases, this component accounts for more than 85% of the total uncertainty budget. This suggests that low-cost sensors’ primary limitation lies in their temporal stability and sensitivity to varying environmental conditions rather than intrinsic noise.

The accuracy achieved after calibration is sufficient for high-resolution spatial mapping of CO_2_, particularly in urban environments where relative concentration gradients are often more informative than absolute values.

Future work will investigate long-term sensor stability under real operating conditions, as well as the selectivity of different low-cost sensor types with respect to cross-sensitivity to other gases and environmental interferents.

Overall, this work demonstrates that, when integrated into a rigorous metrological framework, low-cost gas sensors can provide reliable and scalable tools for atmospheric monitoring. The proposed calibration methodology provides a practical, reproducible basis for characterizing low-cost sensors that target a broad range of atmospheric trace gases.

## Figures and Tables

**Table 1:** List of the most popular commercial low-cost NDIR considered.

Producer	Model	Accuracy	Range (µmol/mol)	Integrated Sensors	Note
Sensirion (Stäfa, Switzerland)	SCD30	±(30 µmol/mol + 3%)	0–40,000	*T*, *RH*	more stable
Sensirion	SCD40	±(40 µmol/mol + 5%)	0–5000	*T*, *RH*	slower
Sensirion	SCD41	±(40 µmol/mol + 5%)	0–5000	*T*, *RH*	smaller, more efficient
Winsentech (Zhengzhou, China)	MH-Z19	±(50 µmol/mol + 5%)	0–5000		cheap but less stable
SenseAir (Delsbo, Sweden)	S8	±(40 µmol/mol + 3%)	0–5000		industrial grade
SenseAir	K30	±30 µmol/mol	0–10,000	optional	more professional
SenseAir	LP8	±(50 µmol/mol + 5%)	0–2000	*T*	based on duty-cycle

**Table 2:** Average CO_2_ values over a 10 min interval for each reading collected.

	Repetitions	(µmol/mol)	(µmol/mol)	(µmol/mol)	(µmol/mol)	(µmol/mol)	(µmol/mol)	(µmol/mol)
**CO_2,ref_**	I	348.02	453.43	551.06	649.26	754.04	850.57	951.46
	II	348.28	453.56	550.77	649.42	754.02	851.36	951.84
	III	349.13	453.64	551.35	649.53	753.93	851.34	952.27
**CO_2,sen1_**	I	319.76	426.77	520.96	592.16	714.06	788.37	866.49
	II	348.79	432.72	515.78	599.60	698.85	826.38	877.96
	III	312.84	423.89	513.56	616.98	682.28	764.57	890.39
**CO_2,sen2_**	I	343.94	443.06	550.28	629.26	734.24	796.89	888.29
	II	355.89	445.26	532.22	599.82	723.00	838.58	883.78
	III	377.59	437.10	531.10	627.87	759.63	854.99	972.49
**CO_2,sen3_**	I	372.31	473.78	577.68	641.29	765.82	836.58	927.47
	II	383.21	472.39	571.22	644.73	751.66	877.87	936.88
	III	352.88	459.84	550.82	645.33	735.78	828.21	952.29

**Table 3:** Average CO_2_ values of the three repetitions and corresponding errors.

	(µmol/mol)	(µmol/mol)	(µmol/mol)	(µmol/mol)	(µmol/mol)	(µmol/mol)	(µmol/mol)
**CO_2,ref_**	348.48	453.54	551.06	649.40	754.00	851.09	951.86
**CO_2,sen1_**	327.13	427.79	516.77	602.91	698.40	793.10	866.49
*ε*_sen__1_	−21.34	−25.75	−34.30	−46.49	−55.60	−57.98	−73.58
**CO_2,sen2_**	359.14	441.81	537.87	618.98	738.96	830.15	914.85
*ε*_sen2_	10.67	−11.74	−13.19	−30.42	−15.04	−20.94	−37.01
**CO_2,sen3_**	369.47	468.67	566.57	643.78	751.09	847.56	938.88
*ε*_sen3_	20.99	15.13	15.51	−5.62	−2.91	−3.53	−12.98

**Table 4:** Uncertainty budget table for sen1.

CO_2,sen1_ (µmol/mol)	*ε*_sen1_ (µmol/mol)	*u*_cal_ (µmol/mol)	*u*_res_sen1_ (µmol/mol)	max *u*_repeat_sen1_ (µmol/mol)	*u*_reprod_sen1_ (µmol/mol)	*u*_c_sen1_ (µmol/mol)	*U*_sen1_ (µmol/mol)
327.13	−21.34	0.70	0.01	0.87	19.08	19.11	38.22
427.79	−25.75	0.90	0.01	0.62	4.50	4.63	9.26
516.77	−34.30	1.10	0.01	0.70	3.80	4.01	8.03
602.91	−46.49	1.30	0.01	0.75	12.74	12.82	25.65
698.40	−55.60	1.50	0.01	0.59	15.89	15.98	31.95
793.10	−57.98	1.70	0.01	0.56	31.18	31.23	62.45
878.28	−73.58	1.90	0.01	0.55	11.95	12.16	24.32

**Table 5:** Uncertainty budget table for sen2.

CO_2,sen2_ (µmol/mol)	*ε*_sen2_ (µmol/mol)	*u*_cal_ (µmol/mol)	*u*_res_sen2_ (µmol/mol)	max *u*_repeat_sen2_ (µmol/mol)	*u*_reprod_sen2_ (µmol/mol)	*u*_c_sen2_ (µmol/mol)	*U*_sen2_ (µmol/mol)
359.14	10.67	0.70	0.01	0.49	17.06	17.08	34.16
441.81	−11.74	0.90	0.01	0.47	4.22	4.34	8.68
537.87	−13.19	1.10	0.01	0.61	10.76	10.83	21.67
618.98	−30.42	1.30	0.01	0.53	16.61	16.67	33.34
738.96	−15.04	1.50	0.01	0.40	18.77	18.83	37.66
830.15	−20.94	1.70	0.01	0.70	29.95	30.01	60.02
914.85	−37.00	1.90	0.01	0.90	49.96	50.01	100.01

**Table 6:** Uncertainty budget table for sen3.

CO_2,sen3_ (µmol/mol)	*ε*_sen3_ (µmol/mol)	*u*_cal_ (µmol/mol)	*u*_res_sen3_ (µmol/mol)	max *u*_repeat_sen3_ (µmol/mol)	*u*_reprod_sen3_ (µmol/mol)	*u*_c_sen3_ (µmol/mol)	*U*_sen3_ (µmol/mol)
369.47	20.99	0.70	0.01	0.65	15.37	15.40	30.79
468.67	15.13	0.90	0.01	0.49	7.68	7.75	15.49
566.57	15.51	1.10	0.01	0.38	14.02	14.07	28.14
643.78	−5.62	1.30	0.01	0.44	2.18	2.58	5.15
751.09	−2.91	1.50	0.01	0.95	15.03	15.13	30.27
847.56	−3.53	1.70	0.01	0.44	26.59	26.64	53.29
938.88	−12.98	1.90	0.01	0.56	12.53	12.68	25.37

**Table 7:** Correction table for all three low-cost sensors.

CO_2_ Concentration Interval (µmol/mol)	*ε*_sen1_ (µmol/mol)	*ε*_sen2_ (µmol/mol)	*ε*_sen3_ (µmol/mol)	*u*_c_sen1_ (µmol/mol)	*u*_c_sen2_ (µmol/mol)	*u*_c_sen3_ (µmol/mol)
300–400	−21.34	10.67	20.99	19.11	17.08	15.40
400–500	−25.75	−11.74	15.13	4.63	4.34	7.75
500–600	−34.30	−13.19	15.51	4.01	10.83	14.07
600–700	−46.49	−30.42	−5.62	12.82	16.67	2.58
700–800	−55.60	−15.04	−2.91	15.98	18.83	15.13
800–900	−57.98	−20.94	−3.53	31.23	30.01	26.64
900–1000	−73.58	−37.00	−12.98	12.16	50.01	12.68

**Table 8:** Results of the straight-line analysis curves fitted by the WTLS method.

	*y = a + bx*					
	*a* (µmol/mol)	*b*	*u*(*a*) (µmol/mol)	*u*(*b*)	*u*(*a*,*b*) (µmol/mol)	*χ*^2^*/*(*n − p*)
**sen1**	−18.87	1.10	14.41	0.02	−0.38	0.03
**sen2**	−11.93	1.05	20.43	0.04	−0.84	0.35
**sen3**	−43.64	1.07	17.79	0.02	−0.49	0.65

## Data Availability

The authors declare that the data supporting the findings of this study are available within the paper. Should any raw data files be needed in another format, they are available from the corresponding author upon request.

## Supplementary Materials

The following supporting information can be downloaded at: https://www.mdpi.com/article/10.3390/s26123685/s1, Figure S1: Photo of the isolator designed at INRiM (Montepaone, Italy); Figure S2: Comparison of CO2 data acquisition of the three low-cost sensors and the reference instruments LI-cor1 and LI-cor2; Figure S3: Temperature and relative humidity measured during the 10 min intervals of analysis; Figure S4: Comparison between the three readings made by sen3 and the three readings made by the reference instrument LI-cor1 for all CO2 ranges analyzed; Figure S5: Comparison of the average of the three readings made by the three low-cost sensors and the reference instrument for all CO2 range analyzed; Figure S6: Percentage of influence of uncertainty components considered for the evaluation of the combined uncertainty for the low-cost sensors: (a) sen1; (b) sen2; (c) sen3; Figure S7: The mean error among repetitions and the expanded uncertainty band ± *U*; Figure S8: Analysis curve for sen2 with associated uncertainty, elaboration results plot Model 3a CCC software; Figure S9: Analysis curve for sen3 with associated uncertainty, elaboration results plot Model 3a CCC software; Figure S10: Analysis of fit errors for sen1 Model 3a across seven distinct measurements; Figure S11: Analysis of fit errors for sen2 Model 3a across seven distinct measurements; Figure S12: Analysis of fit errors for sen3 Model 3a across seven distinct measurements.

## Abbreviations

The following abbreviations are used in this manuscript:

NDIR	Non-Dispersive Infrared Absorption Method
IoT	Internet of Things
CRDS	Cavity Ring-Down Spectroscopy
GAW	Global Atmosphere Watch
WMO	World Meteorological Organization
RMSE	Root Mean Square Error
MAE	Mean Absolute Error
INRiM	Istituto Nazionale di Ricerca Metrologica (IT)
PMMA	Poly(methyl methacrylate)
MFC	Mass Flow Controller
SCCM	Standard cubic centimetres per minute
WTLS	Weighted Total Least Squares method
LoA	Limits of Agreement
T	Temperature
RH	Relative Humidity
LR	Linear Regression
