# Supplementary material for: Metrological Characterization of Low-Cost CO2 Sensors for Environmental Monitoring Applications
Source: Sensors (Basel). 2026 Jun 9;26(12):3685. doi: 10.3390/s26123685 (PMC13306425; doi:10.3390/s26123685)
Supplement: Supplementary file 1 [file sensors-26-03685-s001.zip › sensors-4319787-supplementary.pdf]

Supplementary materials

Metrological Characterization of Low-Cost CO_2 Sensors for Environmental Monitoring Applications

Ramona Russo [1,2], Francesca Rolle [1], Giuliano Vitali [3], Francesca Durbiano [1], Francesca Romana Pennecchi [1], Stefano Pavarelli [1] and Michela Sega [1,*]

[1] Istituto Nazionale di Ricerca Metrologica (INRiM), 10135 Torino, Italy; r.russo@inrim.it (R.R.); f.rolle@inrim.it (F.R.); f.durbiano@inrim.it (F.D.); f.pennecchi@inrim.it (F.R.P.); s.pavarelli@inrim.it (S.P.)

[2] Department of Energy (DENERG), Politecnico di Torino, 10129 Torino, Italy

[3] Department of Agricultural and Food Sciences, University of Bologna, 40126 Bologna, Italy; giuliano.vitali@unibo.it

* Correspondence: m.sega@inrim.it

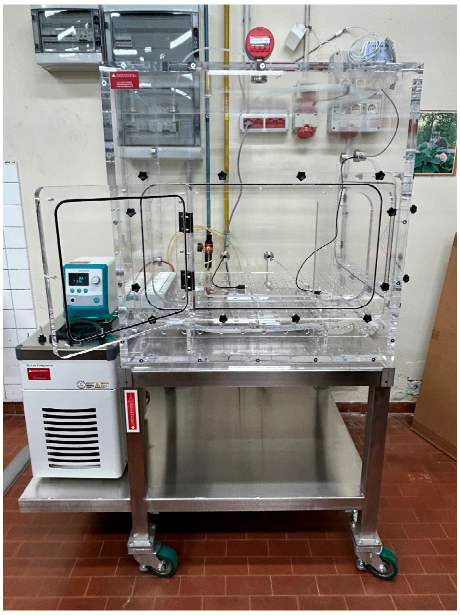

Figure S1. Photo of the isolator designed at INRiM (Montepaone, Italy).

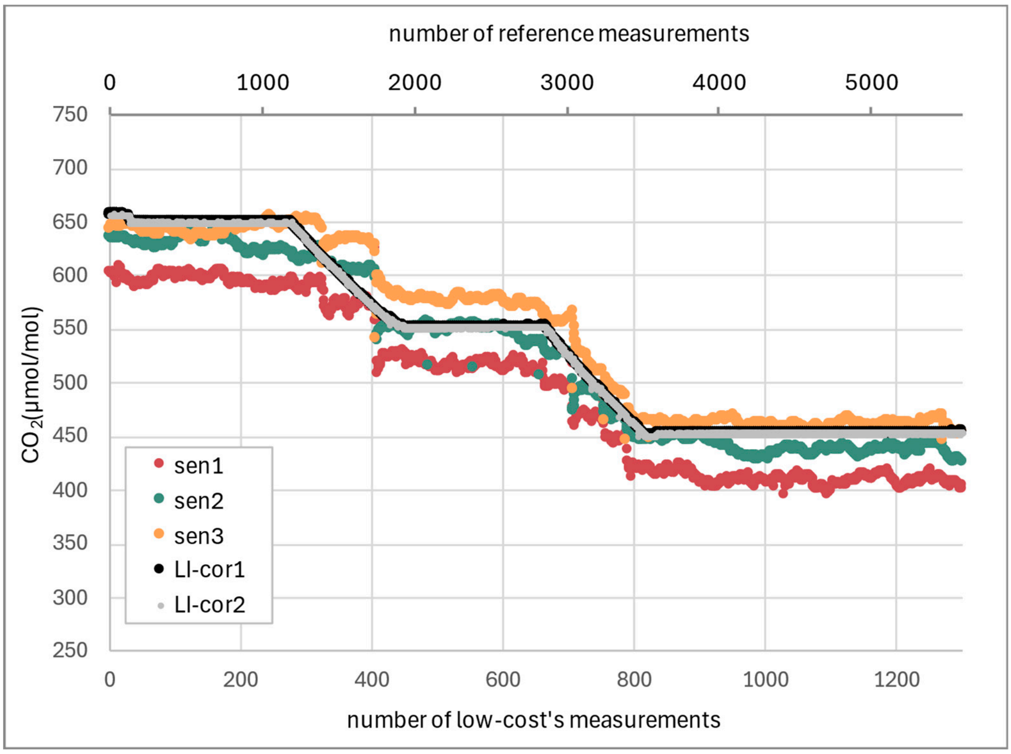

Figure S2. Comparison of CO_2 data acquisition of the three low-cost sensors and the reference instruments LI-cor1 and LI-cor2.

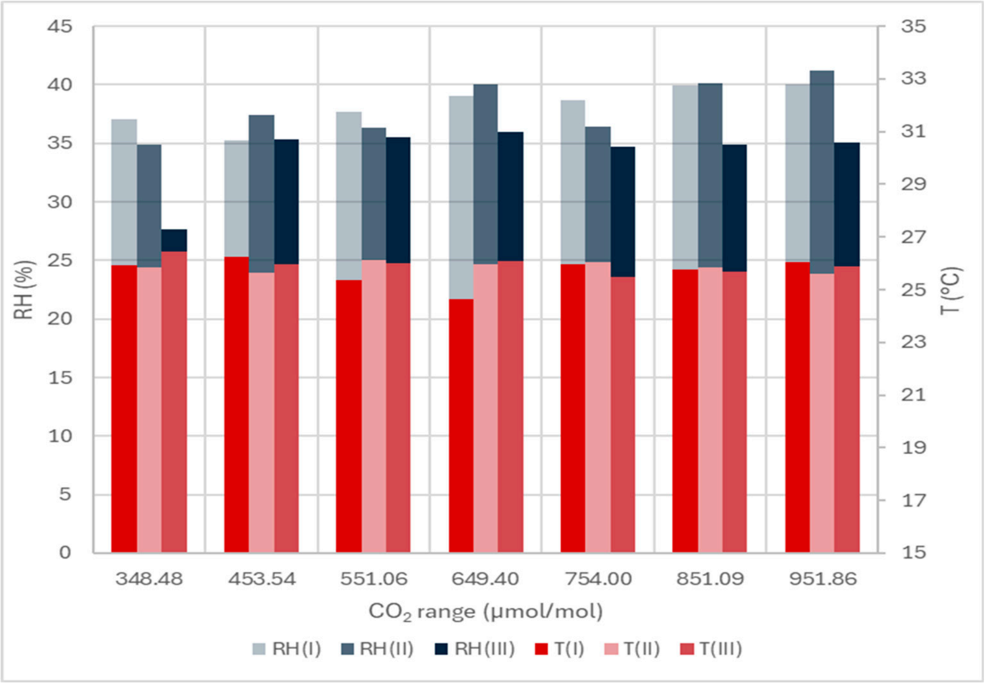

Figure S3. Temperature and relative humidity measured during the 10-min intervals of analysis.

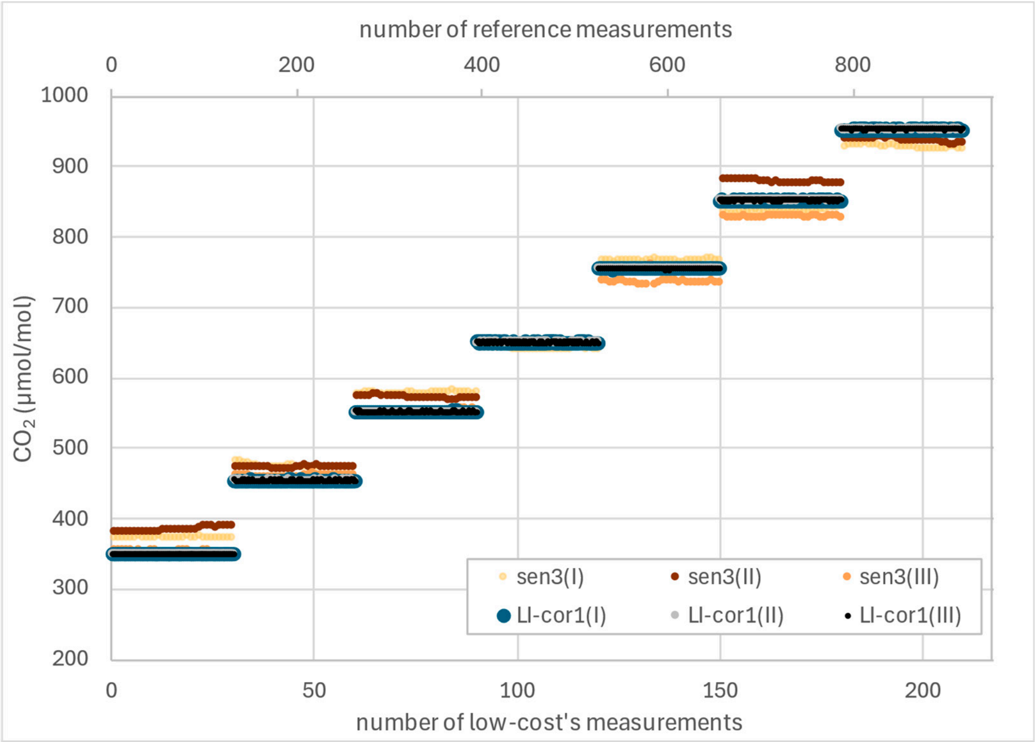

Figure S4. Comparison between the three readings made by sen3 and the three readings made by the reference instrument LI-cor1 for all CO_2 ranges analysed.

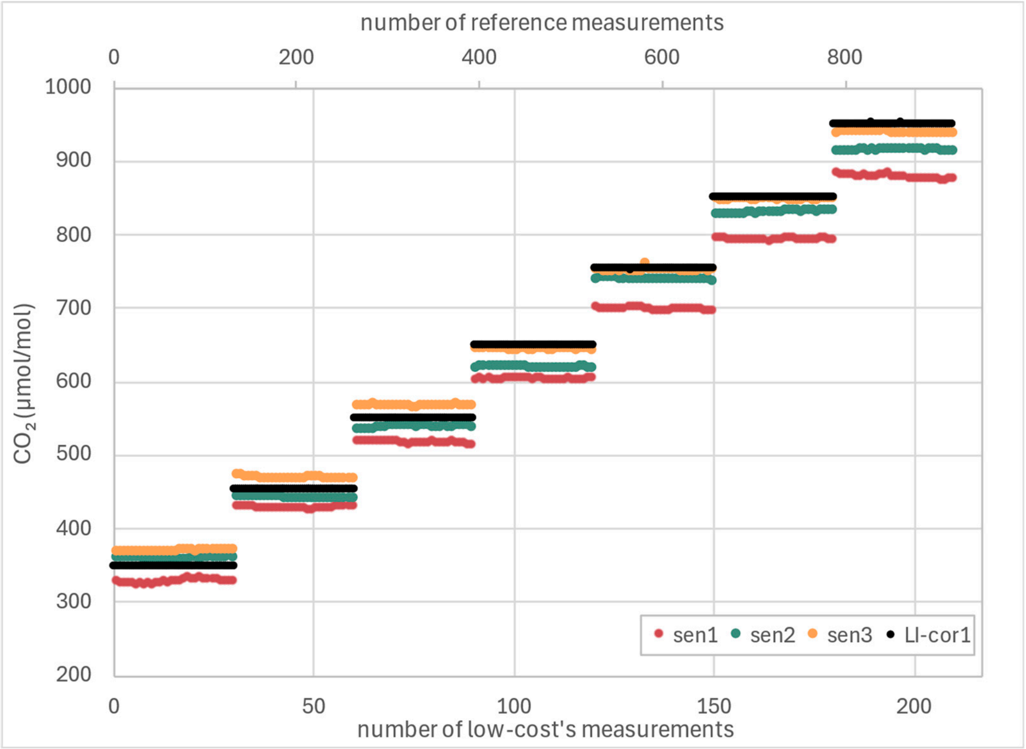

Figure S5. Comparison of the average of the three readings made by the three low-cost sensors and the reference instrument for all CO_2 range analysed.

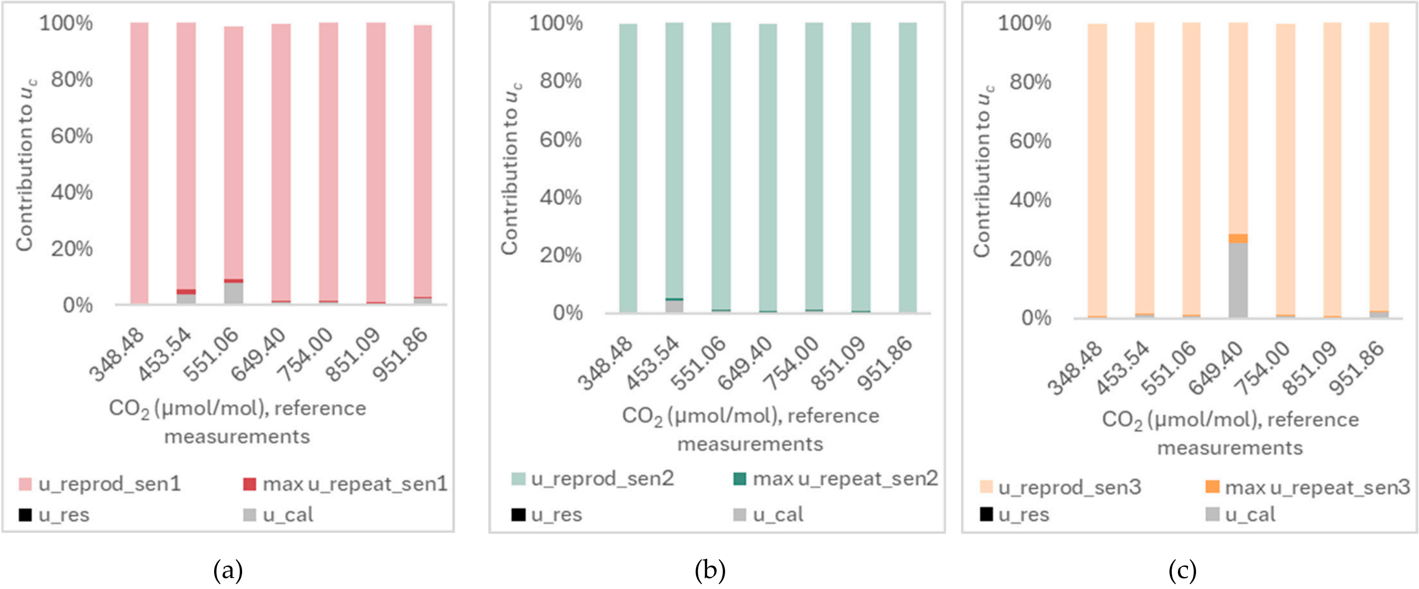

Figure S62. Percentage of influence of uncertainty components considered for the evaluation of the combined uncertainty for the low-cost sensors: (a) sen1; (b) sen2; (c) sen3.

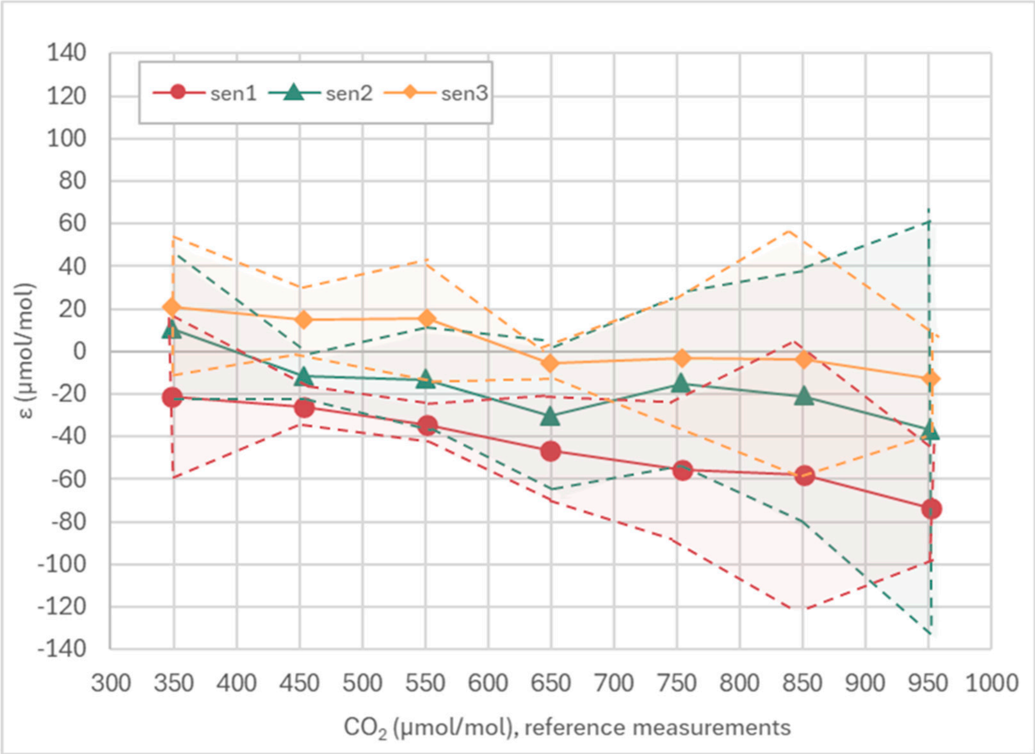

Figure S7. The mean error among repetitions and the expanded uncertainty band ± *U*

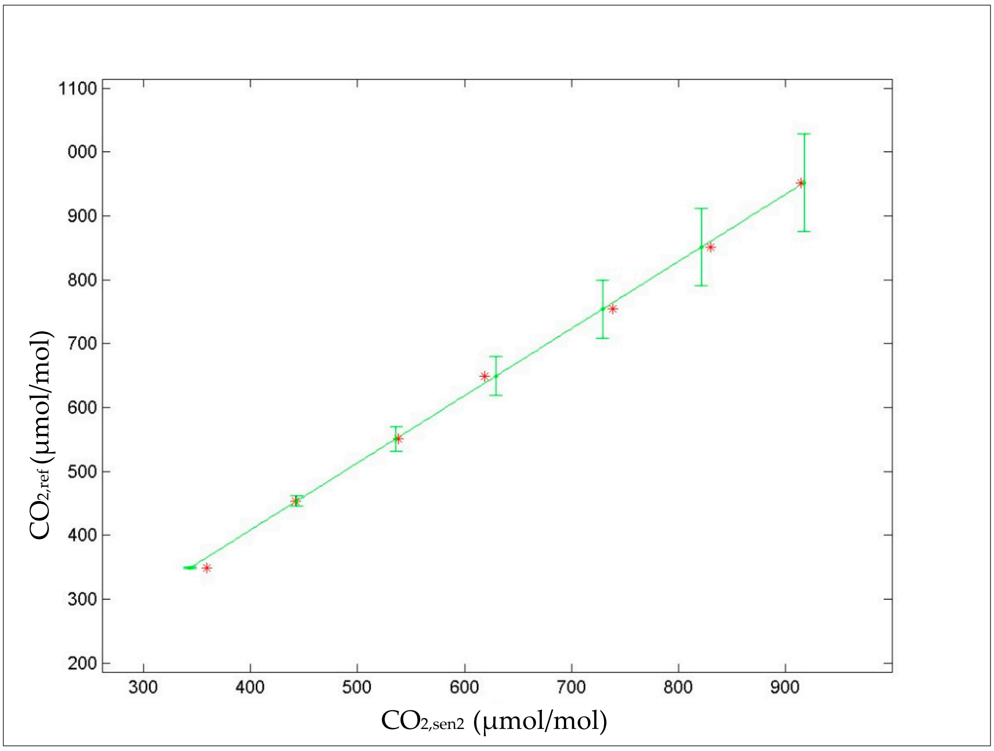

Figure S83. Analysis curve for sen2 with associated uncertainty, **e**laboration results plot produced by the CCC software for a linear WTLS regression.

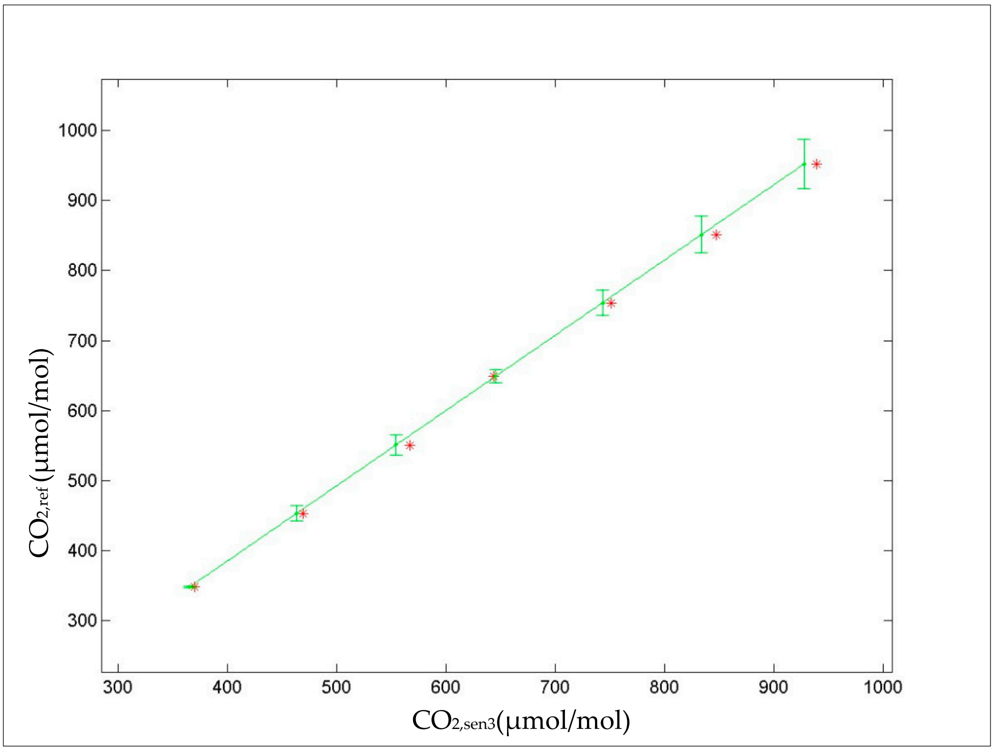

Figure S94. Analysis curve for sen3 with associated uncertainty, **e**laboration results plot produced by the CCC software for a linear WTLS regression.

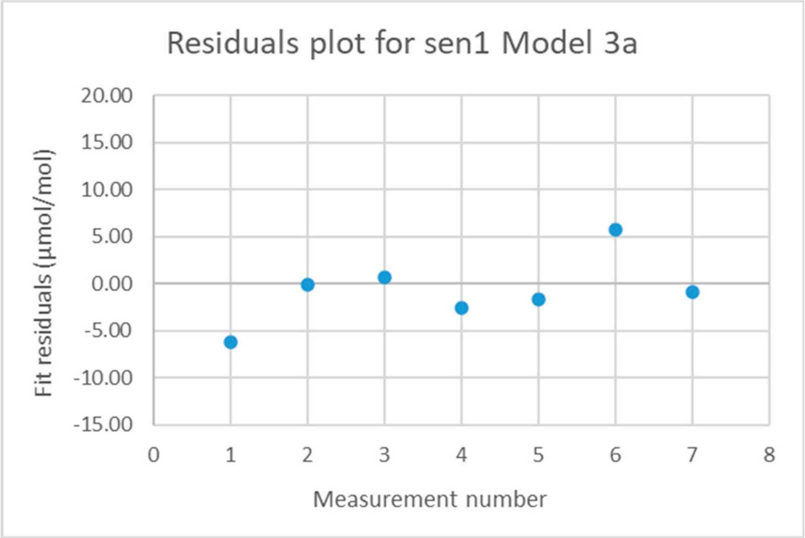

Figure S105. Analysis of fit errors for sen1 Model 3a across seven distinct measurements.

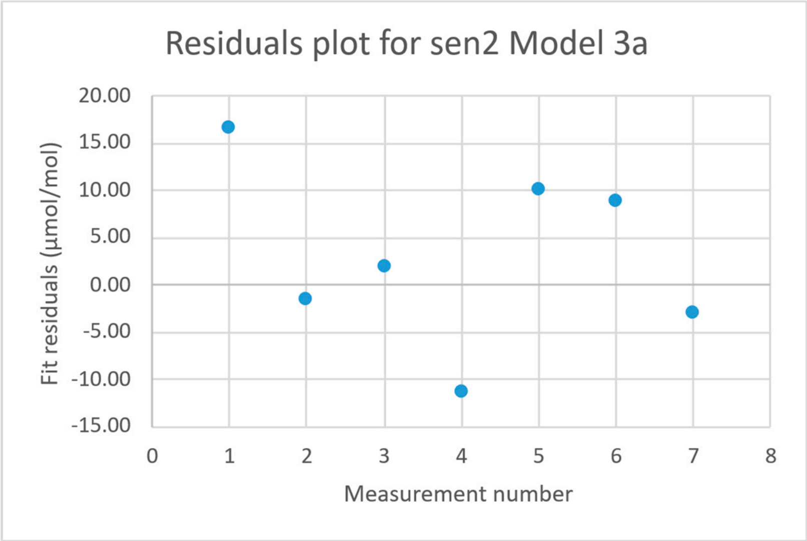

Figure S116. Analysis of fit errors for sen2 Model 3a across seven distinct measurements.

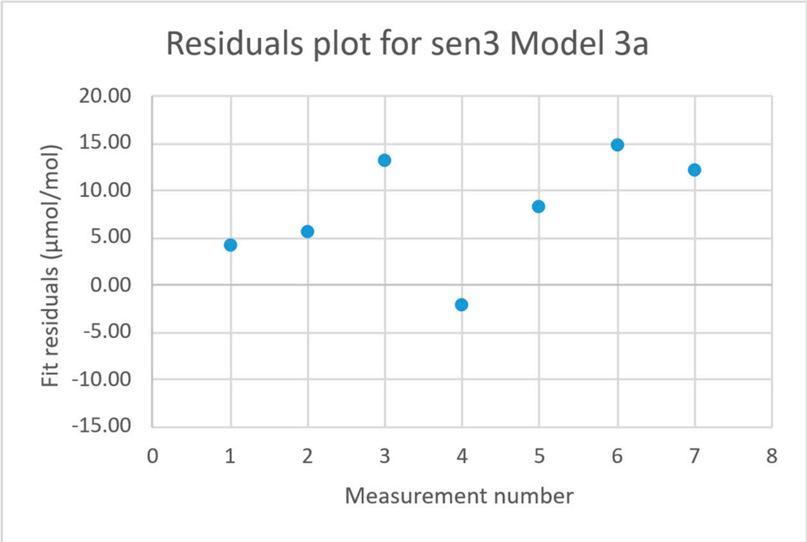

Figure S127. Analysis of fit errors for sen3 Model 3a across seven distinct measurements.
